# Obesity as a Risk Factor for Tendinopathy: A Systematic Review

**DOI:** 10.1155/2014/670262

**Published:** 2014-08-19

**Authors:** Francesco Franceschi, Rocco Papalia, Michele Paciotti, Edoardo Franceschetti, Alberto Di Martino, Nicola Maffulli, Vincenzo Denaro

**Affiliations:** ^1^Department of Orthopaedic and Trauma Surgery, Campus Bio-Medico University of Rome, Via Alvaro del Portillo 200, Trigoria, 00128 Rome, Italy; ^2^Department of Musculoskeletal Disorders, Faculty of Medicine and Surgery, University of Salerno, Baronissi, 84081 Salerno, Italy; ^3^Centre for Sports and Exercise Medicine, Barts and The London School of Medicine and Dentistry, Mile End Hospital, 275 Bancroft Road, London E1 4DG, UK

## Abstract

*Purpose.* In the last few years, evidence has emerged to support the possible association between increased BMI and susceptibility to some musculoskeletal diseases. We systematically review the literature to clarify whether obesity is a risk factor for the onset of tendinopathy. *Methods.* We searched PubMed, Cochrane Central, and Embase Biomedical databases using the keywords “obesity,” “overweight,” and “body mass index” linked in different combinations with the terms “tendinopathy,” “tendinitis,” “tendinosis,” “rotator cuff,” “epicondylitis,” “wrist,” “patellar,” “quadriceps,” “Achilles,” “Plantar Fascia,” and “tendon.” *Results.* Fifteen studies were included. No level I study on this subject was available, and the results provided are ambiguous. However, all the 5 level II studies report the association between obesity measured in terms of BMI and tendon conditions, with OR ranging between 1.9 (95% CI: 1.1–2.2) and 5.6 (1.9–16.6). *Conclusions.* The best evidence available to date indicates that obesity is a risk factor for tendinopathy. Nevertheless, further studies should be performed to establish the real strength of the association for each type of tendinopathy, especially because the design of the published studies does not allow identifying a precise cause-effect relationship and the specific role of obesity independently of other metabolic conditions.

## 1. Introduction

Tendinopathies are common musculoskeletal diseases affecting the tendons. The term tendinopathy describes a range of clinical conditions related to tendons and surrounding structures [1, 2].

Although tendinopathies also include conditions of damage to the tendon in absence of symptoms, these pathologies often occur with pain in the injured tendon, which is accentuated or appears during palpation of the affected area or during active and passive movements involving the tendon.

Pain is often associated with a reduction in the strength of the muscles attached to the tendons involved in the pathological process [3, 4]. Chronic tendinopathies are a common problem for patients whose activities require repetitive movements; for this reason, they are particularly widespread among sportsmen. These conditions can also occur after an acute injury, when the healing process of the injured tendon fails [4]. In the past, the terms “tendinitis” and “tendinosis” were widely and indiscriminately used in place of tendinopathy, often considering this condition as an inflammatory pathology, but such definitions should be imposed only after a histological study [5]. Actually, histological samples from chronic tendinopathies have confirmed that there is no acute inflammatory condition, but rather a failure of the tendon repair associated with angiofibroblastic degeneration [4, 6, 7]. In fact, histologically, the findings are more typical of a “failed healing response,” with a haphazard proliferation of tenocytes, intracellular abnormalities in tenocytes, disruption of collagen fibers, and subsequent increase in noncollagenous matrix.

However, factors that predispose to tendinopathies have not yet been clarified, although there is evidence to support a role for biomechanical factors, functional alterations, aging, and metabolic disorders [7, 8].

Particularly, obesity has recently been indicated as important but potentially modifiable risk factor in the onset and progression of some tendinopathies [9]. In fact, in contrast to other conditions, the advantage from studying obesity lies in the possibility of preventing and treating this risk factor.

Obesity is already a well-known risk factor for many other diseases of the musculoskeletal system [9]. The prevalence of obesity in industrialized countries has increased steadily in recent decades. In the United States, between 2007 and 2008, the prevalence of obesity in adults was estimated to be 32.2% in men and 35.5% in women, reaching percentages of 72.3% in men and 64.1% in women if both obesity and overweight are considered together. In Europe, the prevalence of obesity appears tripled since 1980 and each year four million people become obese [10, 11].

The World Health Organization recommends a standard classification of adult overweight and obesity using the following body mass index (BMI) calculations: a BMI of 25.0 to 29.9 kg per m^−2^ is defined as overweight; a BMI of 30.0 kg per m^−2^ or more is defined as obesity [10, 11].

Other measurements are also used to identify a pathological fat distribution, such as waist circumference (cm) or waist/hip ratio.

The purpose of this review was to summarise the current literature reporting data on the relationship between obesity and tendons diseases to verify the hypothesis that obesity is a risk factor for the development of tendinopathy.

## 2. Methods

**Figure 1:**
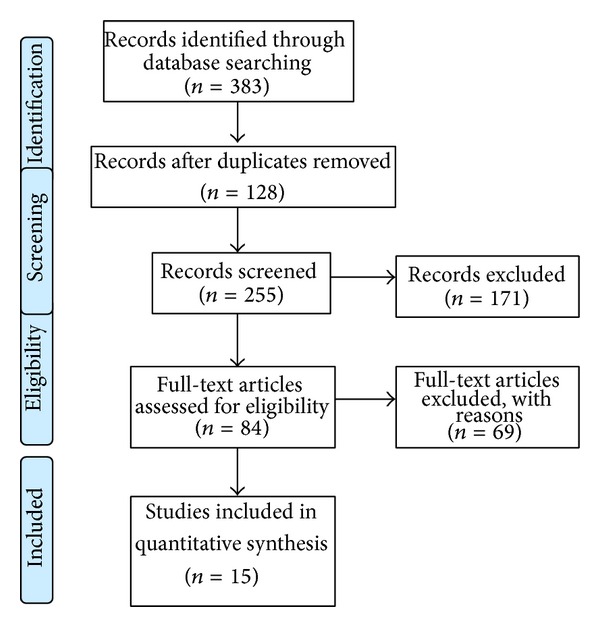
PRISMA 2009 flow diagram.

The systematic review was performed following the PRISMA (preferred reporting items for systematic reviews and meta-analyses) statement [12, 13]. The PRISMA search algorithm is shown in Figure 1.

We searched PubMed, Cochrane Central, and Embase Biomedical databases using the keywords “obesity,” “overweight,” and “body mass index” linked in different combinations with the terms “tendinopathy,” “tendinitis,” “rotator cuff,” “epicondylitis,” “wrist,” “patellar,” “quadriceps,” “Achilles,” “plantar fascia,” and “tendon.” No limit regarding the year of publication and the study design was imposed. We selected articles in English, Spanish, French, and Italian, according to the authors' skills. All peer-reviewed journals were evaluated and all relevant articles were retrieved. Three authors (Francesco Franceschi, Edoardo Franceschetti, and Michele Paciotti) independently reviewed the text of each abstract. Full-text versions were obtained to include or exclude the studies. Clinical studies investigating, as declared aim of the study, the association between obesity and one or more types of tendinopathy were selected. The definition of obesity had to be based on instrumental evaluation through body mass index (BMI) or waist circumference (WC) or waist-to-hip ratio (WHR).  We screened the references lists of the studies found in order to find additional relevant publications.

Demographics data, diagnosis, design of the study, objective means of measuring the weight, and main findings concerning the statistical association between increased weight and tendinopathy were independently extracted by all the investigators.

Biomechanical studies, case reports, literature reviews, technical notes, and instructional courses were excluded. We also excluded articles reporting data of subjects of less than 18 years of age. To avoid bias, all the included articles were reviewed and discussed by all the authors.

## 3. Results

The literature search and cross-referencing resulted in 383 references, of which 299 were rejected due to off topic abstract and/or duplication of the results (Figure 1). After reading the remaining full-text articles, another 69 articles were excluded for failing to fulfil the inclusion criteria. The remaining 15 articles, including 5 frequency-matched case-control studies [14–18], 4 cross-sectional studies [19–22], 5 retrospective case-control studies [23–27], and 1 case-series study [28], were included in the present study.

The total number of patients in the included studies was 36,843, of which 9,002 were the subjects affected by tendinopathy.

All the characteristics of the studies are shown in Table 1.

## 4. Discussion of the Results

In this study, we reviewed all the data provided by published studies that focused on analysing the association between obesity and the development of the most frequent kind of tendinopathy.

### 4.1. Obesity and Rotator Cuff Tendinopathy

Rotator cuff (RC) tendinopathy is most frequently observed.

Rotator cuff disease, which includes a range of clinical and pathological characteristics, is a multifactorial condition, the origin of which is unclear, but the failed healing response typically seen in other tendinopathies is the end result [29]. In fact, the theory, common in the past, based on the mechanical impingement of the rotator cuff has not been demonstrated and does not explain the clinical manifestations of the pathology.

Several studies tried to identify the risk factors involved in this condition.

Some studies suggested a link between shoulder disorders and metabolic factors, such as diabetes mellitus [11].

Diabetes was clearly demonstrated to be associated with RC tendinopathy, increasing incidence and affecting postinjury healing process [30]. Regarding obesity, we did not find the same amount of evidence.

In particular, there are very few studies in literature; none of them is a level I study, and each study has a different design.

The prognostic study performed by Wendelboe et al. [14] in 2004, analysing 311 participants, showed that individuals with a BMI ≥35.0 had an increased risk to require rotator cuff repair with an odd ratio of 3.1 (CI 1.3–7.6) for males and 3.5 (1.8–6.9) for females. Moreover, this risk was directly correlated with the grade of obesity for both men (*P* = .002) and women (*P* < .001).

In 2010, Rechardt and colleagues [19] carried out a cross-sectional study investigating the national Finnish Health Survey. They evaluated if smoking, waist circumference, and waist-to-hip ratio were related to an increased prevalence of shoulder pain in both men and women. Metabolic syndrome, type 2 diabetes mellitus, and carotid intima-media thickness were associated with shoulder pain in men, whereas high level of C-reactive protein was associated with shoulder pain in women. Increased waist circumference and type 1 diabetes mellitus were associated with chronic rotator cuff tendinitis in men.

A large case-control study was performed by Titchener et al. [23] using The Health Improvement Network database to assess and to quantify the relative contributions of some constitutional and environmental risk factors for rotator cuff disease in the community. Their data included 5000 patients with rotator cuff disease who were individually matched with a single control by age, sex, and general practice (primary care practice). Multivariate analysis showed that only “overweight” body mass index of 25.1 to 30 (OR = 1.15) was significantly associated with rotator cuff disease, and, contrarily, mass index greater than 30 was not found to be associated with rotator cuff disease.  However, the authors declared the impossibility to differentiate comorbid factors such as diabetes mellitus, atherosclerosis, and hyperlipidemia.

### 4.2. Obesity and Elbow Tendinopathies

Lateral and medial epicondylitis, also known as “tennis elbow” and “golf elbow,” respectively, are the most common tendinopathies of the elbow. They are pathological conditions of the proximal insertion of the forearm muscles at the humeral epicondyles, which mostly involve the common wrist extensor muscle (lateral epicondylitis) and the common wrist flexor muscle (medial epicondylitis) [20].

The cause of epicondylitis is unknown; it is hypothesized that the lesions occur because of a combination of mechanical overloading and abnormal microvascular responses. Consequently, also the risk factors for this pathology are not well identified.  In 2013, Titchener and coworkers [24] matched 4998 participants with controls to evaluate different environmental and constitutional risk factors for epicondylitis. Their results showed that patients with a BMI over 40 were at higher risk of being affected by lateral epicondylitis than those with normal BMI [OR 1.41 (1.01–1.97)]. However, this association disappeared when BMI was adjusted for consultation rate using multivariate conditional logistic regression [OR 0.94 (0.66–1.34)].

In the level IV study performed by Descatha and colleagues [28], designed to assess the incidence of epicondylitis in workers exposed physically, the stratification of the risk factors, made by univariate analysis, showed how subjects with BMI >30 kg/m^2^ had higher incidence rates for the disease (OR: 2.4, CI: 1.2–4.8). No significant association was instead found for medial epicondylitis.

Conversely, the cross-sectional study by Shiri et al. [20], developed to primarily investigate the prevalence and the risk factors associated with lateral and medial epicondylitis, assessed a causal relationship only between medial epicondylitis in women and both waist circumference >100 cm (OR: 2.7 CI: 1.2–6.0) and BMI >30 kg/m^2^ (OR: 1.9 CI: 1.0–2.7), with no increased risk as regards lateral epicondylitis.

### 4.3. Obesity and Knee Tendinopathies

Knee pathologies such as arthritis are known to be particularly common among obese patients [31, 32].

Regarding knee tendinopathies, a nosological distinction should be made between extensor apparatus tendinopathies and pes anserinus tendinopathies. Diseases of the extensor apparatus, commonly observed among sportsmen, affect quadriceps tendon and patellar tendon at their bony attachments. Pes anserinus tendinopathies are characterized by the presence of pain under load, standing, walking, or taking the stairs, at the insertion of muscles semimembranosus, semitendinosus, gracilis, and sartorius in the superomedial surface of the tibia. It is very frequent in obese women with valgus knee because the hamstring tendons rub against the medial condyle of the knee during every movement. The anserine bursa, which lies between the tendons footprint and the posterior surface of the tibia, may be involved in the inflammatory process and leads to the so-called anserine bursitis which is part of the tendinopathy [33].

A case-control study by Alvarez-Nemegyei [25], performed in 2007 and involving 22 cases and 38 controls, failed to find a relationship between pes anserinus tendinitis/bursitis and diabetes or obesity.

Likewise, Taunton et al. [27], retrospectively analysing 96 cases of patellar tendinopathy among a total of 2002 running related injuries, found neither an increased weight nor an increased BMI in those kinds of patients.  Panasiuk and Groblewski [34] presented a case report on a patient with BMI =41, without other comorbidities, who atraumatically injured his patellar tendon. According to the authors, the increased load played a crucial role as cofactor in the mechanism of this spontaneous tendon rupture.

They underlined the dangerousness of such a high load and how individuals with important obesity have a potential risk of acute tendon injuries due to the increased weight and mass.

In literature, there are other case reports [35, 36] dealing with the spontaneous ruptures of patellar tendon or quadriceps tendon, and an increased weight of the patient is often reported among the risk factors.

However, given the rarity of these conditions, no sufficient clinical studies of high level of evidence have been made on this topic; therefore, there are no statistically valid information on the possible association between obesity and quadriceps tendon rupture.

### 4.4. Obesity and Achilles Tendinopathy

Micro-traumatic tendinopathies of the Achilles tendon are functional overload pathologies that can lead to rupture of the tendon, the ultimate result of a long standing process of failed healing response. In Achilles tendon rupture patients, this failed healing response process is most often entirely asymptomatic, and, involving the tendon in variable extension, determines a decrease in mechanical strength of the tendon, which can be overcome by a sudden strain, and result in a tear [37]. Holmes and Lin [26] in 2006 studied some metabolic risk factors (obesity, diabetes, hypertension, use of oestrogen, and exposure to steroids) to define and quantify their possible etiological role in Achilles tendinopathy.

Using Chi-square analysis to compare observed and expected prevalence in a group of 82 participants versus published national data, they found a statistically significant association for all these conditions and Achilles tendinopathy. In particular, as regards obesity, it was associated with Achilles tendinopathy for both men and women subjects (*P* = .001 and .0025). Since the microcirculation is the common denominator between all of these metabolic diseases, alterations of blood flow were suspected to underlie the onset of Achilles tendinopathy.

A 2007 cross-sectional study by Frey and Zamora [21] showed a high BMI (both in overweight and in obese range) significantly increased the chances of Achilles, posterior tibial, and peroneal tendinitis. In particular, 123 (65.4%) of the overweight/obese subjects had a diagnosis of tendinitis compared to 65 (34.6%) normal subjects, and having a BMI >25 increases the risk of being affected by tendinitis [OR: 1.923 (1.39–2.66) *P* <  .0001].

Also, Gaida and coworkers in 2010 [22] investigated the relationship between adiposity and asymptomatic Achilles tendinopathy through a cross-sectional study. Examining 298 individuals, they found that men with Achilles tendon pathology had a central fat distribution, while women with tendon pathology had a peripheral fat distribution. These opposite findings, seemingly paradoxical, according to the authors depend on the effect that oestrogens have on the deposition of fat in women. They concluded that the asymptomatic condition of the participants is a clear and important proof that differences in adipose tissue distribution precede tendon pain. In 2013, Scott and colleagues [15] compared 197 patients affected by Achilles tendinopathy versus 100 controls to investigate the relationship between Achilles tendinopathy and body mass index. They found a statistically significant difference in terms of BMI (34.69 ± 7.54 (17.9–75.9) versus 30.56 ± 7.55 (19.7–61.5), *P* < .001) and mean age between the two groups.

Similarly, in the 10-year retrospective analysis performed by Klein et al. [16] on 944 subjects, mean BMI was significantly higher in the group of patients with Achilles tendonitis compared to the control group (30.2 ± 6.5 versus 25.9 ± 5.3, *P* < .001). Overweight and obese patients were 2.6 to 6.6 times more likely than patients with normal BMI to be affected by Achilles tendonitis (*P* < .001).

Taunton et al. [27] carried out a retrospective case-control analysis of 2002 running related injuries. Comparing the 96 cases of Achilles tendinopathy they recorded, with the other 1906 patients, they found no statistically significant association between obesity and this tendinopathy.

### 4.5. Obesity and Plantar Fasciitis

It is intuitive to speculate that an excess of body weight may be a determinant factor in the common feet pain. In obese subjects, the baropodometric examination reveals very often the loss of the transverse foot arch, resulting in discharging the body weight on the central metatarsal heads with pain on walking [38]. Riddle and colleagues [18], in their level II prognostic study, matched 50 patients affected by plantar fasciitis with 100 controls. They obtained that participants with a BMI >30 kg/m^2^ are 5.6 times (CI: 1.9–16.6) more likely to be affected when compared with subjects with BMI ≤25 kg/m^2^.  Taunton et al. [27] carried out a retrospective case-control analysis of 2002 running related injuries and reported that a high body weight in women (>60 kg) was associated with plantar fasciitis (OR: 0.378, CI: 0.203–0.706).

The Australian group of Frey and Zamora [21], in 2007, identified obesity (along with the pronated foot) as independent and modifiable risk factor for chronic plantar heel pain, through a univariate analysis performed on 80 patients and 80 controls.

Considering that this study cannot establish causality, it is unclear whether increased BMI existed in the case group participants prior to the development of CPHP or whether the pain associated with the condition caused participants to reduce their physical activity, thereby leading to an increase in BMI. However, it is plausible that increased BMI may be a risk factor for CPHP as individuals with increased BMI experience higher vertical forces under the heel during gait [39], leading to higher internal stresses within the heel [40], which may lead to damage of soft tissue structures and the development of symptoms.

An increased incidence of chronic plantar heel pain in individuals with a BMI >25 Kg/m^−2^ was also demonstrated by a study by Irving et al. [17]. They also found an increased chance, although not significant, to be affected by plantar fasciitis if overweight or obese. The authors proposed to relate these data to the effect of an increased weight on musculoskeletal disorders of the lower district (feet and ankles), which are known to be caused by overuse and stress, which are factors made worse by weight.

A recent review [41] established that adult obese individuals are three times more affected by chronic plantar heel pain and foot pain compared to normal weight subjects.

This association includes plantar fasciitis, a condition closely associated with obesity. However, the authors did not exclude the existence of a reverse causality, whereby the presence of plantar pain intervenes by limiting the mobility, thus favouring being overweight. Another discussion point involved the effectiveness of weight loss on the pain symptoms reduction. In fact, the studies reviewed did not provide evidence of a recovery from the distressing symptoms following bariatric surgery or other weight-loss strategies.

### 4.6. Comments

Most of the published articles that we analysed in this review are observational studies. However, to date, no level I study was performed about this topic. All the 5 frequency-matched case-control studies (level II), 14–18 published on this matter, agree to report the association between obesity measured in terms of BMI (BMI ≥ 30 kg/m^2^) and tendon diseases, with odds ratios ranging from 1.9 (95% CI: 1.1–2.2) to 5.6 (1.9–16.6).   All the 4 cross-sectional studies, included in this review, also indicate an association between these two conditions, with the exception of the part of the study by Frey and Zamora [21] concerning plantar fasciitis, which however finds a correlation, although not significant.

Nevertheless, such study designs do not allow a precise identification of a cause-effect relationship between pathological body mass index and each type of tendinopathy. At present, in fact, not enough works focused on the mechanism through which excess weight may be responsible for tendinopathies.  The largest amount of studies investigating pathophysiological mechanisms focused on Achilles tendinopathy. A 2013 murine study by Boivin et al. [42] examined both the potential negative effect of obesity on Achilles tendon and quadriceps muscle and the potential mitigating effect of exercise and branched-chain amino acid (BCAA) on the same structures. After subjecting the mice to a high fat diet (and its resulting obesity), they found significant alterations in the structure of the Achilles tendon removed from the mouse, with increased tendon cross-sectional area and decreased modulus. Exercises and BCAA integration improved only partially the outcomes, decreasing the stiffness of the Achilles tendon. The authors speculated that the exceeding fat intake, causing the enlargement of the diameter of the fibers and the shortening of the modulus of the tendon, actually leads to a stiffer tendon, less able to withstand the loads.

In 2012, another study [43] focused attention on the Achilles tendon, using 20 healthy adult males divided into low normal weight and overweight based on BMI. The authors measured, by ultrasound, the thickness of the Achilles tendon before and after a session of calf training with ankle weights on. Their aim was to assess the cumulative transverse tendon strain defined as the natural log of the ratio of post- to preexercise tendon thickness. While the thickness, in absolute terms, was greater, both before and after training, for the group classified as “overweight,” in fact, the acute transverse strain response was significantly higher in the group of healthy subjects (−11% versus −20%, *P* = .0004). Their pathophysiological explanation of the obtained findings is based on the harmful effect that the tensile load exerts on cell matrix and particularly on morphology and disposition of collagen fibers; this would alter the physiological movement of interstitial fluids, not allowing a proper and normal response to exercise.

A similar study was carried out by Abate et al. [3] in 2012 recruiting a sample of athletes (runners) and a control sample of nonrunning subjects and dividing both groups further into two groups: normal weight and overweight. The results obtained by US (ultrasound) showed a statistically significant difference in terms of the thickness of the Achilles tendon only between runners and nonrunners among normal weight subjects (*P* = .002), indicating that the physiological hypertrophy of the tendon occurs only in normal subjects. Conversely, both US abnormalities and intratendinous microvessels were observed more frequently in overweight participants tendons (*P* = .0007 and *P* = .0003) and, within this group, were significantly prevalent in runners (*P* = .001 and *P* = .004). The authors attribute these findings to the significantly lower ability of the tendon of an obese subject to resist the stress (such as running) and to repair the damage caused by the stress.  According to some other hypothesis, a prolonged state of systemic, low-grade inflammation, such as in obesity and states of impaired insulin sensitivity, may act as a risk factor for a “failed healing response” after an acute tendon insult, thus predisposing affected individuals to development of chronic overuse tendinopathies [1, 6].  However, it should be necessary to distinguish what could be the real burden of obesity in the pathophysiological process that leads to tendinopathy. Obesity is present in a number of metabolic diseases such as diabetes which have been associated with tendinopathy on cardiovascular grounds, not the obesity per se. It is known that obesity is associated with alterations in glucose metabolism and conditions as dyslipidemia, hypertension, glucose intolerance, and insulin resistance. These were found both in obese patients and in patients affected by tendinopathy [44].

Future studies should carry out clinical observations on obese patients affected by tendinopathy, distinguishing when obesity is associated with other metabolic diseases and when it is not.

In addition to metabolic pathophysiological mechanisms, mechanical factors are supposed to play a role in the onset of tendinopathy. In particular, among the studies evaluated in this review, there is a stronger association between lower limb tendinopathies and obesity, compared to upper limbs, which seems to prove the hypothesis that higher loading force can be an important risk factor.

Our results are consistent with those obtained from the systematic review performed by Gaida et al. [44] in 2009, which analysed studies published until March 2007. By means of the sensitive analysis, they found 81% of positive association between increased adiposity and tendinopathies considering trials including clinical patients and 77% considering case-control studies.

They also reported poorer outcomes among obese individuals after the treatment of a tendon injury.

Analysing the long-term results and effects of a pathological BMI on the tendinopathy healing process and on the surgical outcomes is certainly an area of research that can provide useful findings, especially if further research will be particularly focused on the differences in results between subjects with obesity compared to those affected by multiple metabolic diseases.

## 5. Conclusions

Obesity is widespread and, therefore, it is very easy to run into patients with tendinous pathologies who are also overweight.

The best evidence available to date indicates obesity as a risk factor for tendinopathy. In particular, this association seems strong for Achilles tendinopathy and for plantar fasciopathy, in which the increased weight creates an increased load for the tendons, stressing these structures.

Nevertheless, given the low number of high-level studies on the subject, the relationship between obesity and tendinopathies is still enigmatic. Much remains to be studied on this matter and further studies should be performed to establish the real strength of the association between each type of tendinopathy and the obesity per se, isolated from all other metabolic diseases.

Future research will have to go in two directions: clinically, analysing clinical data to confirm and quantify the correlation of obesity with tendinopathies, and experimentally, examining the possible pathophysiological mechanisms underlying this causal relationship.

## Figures and Tables

**Table 1:** Summary of the studies.

Authors	Year	Tendinopathy	Study design	Number of patients	Age in years (range)	Measures of obesity	Relevant results	Conclusions of the study	Association
Wendelboe et al. [14]	2004	Rotator cuff	Frequency-matched case-control study Prognostic study, Level II	311 RCR cases versus 993 controls	(53–77)	BMI	OR: 1.9 (95% CI: 1.1–2.2) for males and 2.4 (1.4–4.2) for females with BMI ≥ 35. OR: 3.1 (95% CI: 1.3–7.6) for males and 3.5 (1.8–6.9) for females with BMI ≥ 35. Risk directly correlated with the grade of obesity for both men (*P* = .002) and women (*P* < .001)	Obesity increases risk to need RCR	Yes

Rechardt et al. [19]	2010	Rotator cuff	Cross-sectional study (population study)	6,237 of which chronic 28 (2.8%) with RC tendinitis	50.8 for men, 52.9 for women (>30)	BMI, waist circumference, waist-to-hip ratio	WC 94.0–101.9 cm and RC tendinitis OR: 2.0 (1.1–3.5) in men	Increased WC is associated with chronic RC tendinitis in men	Partial

Titchener et al. [23]	2014	Rotator cuff	Retrospective case-control, treatment study Level III	5,000 cases of RC disease versus 5,000 controls (BMI calculated in 3,385 of cases (67.7%) and 3,050 (61.0%) of controls)	55 (interquartile range 44–65)	BMI	BMI 25.1–30 (overweight) and RC disease OR: 1.23 (1.10–1.38) BMI 30.1–40 (obese) and RC disease OR: 1.25 (1.09–1.44) BMI > 40 (morbidly obese) no increased risk After adjustment for consultation rate, the effect persisted only in the BMI 25.1–30 (overweight) group OR: 1.15 (1.02–1.31)	Significant association only for patients who are slightly overweight (BMI 25–30) Impossible to differentiate comorbid factors such as diabetes mellitus, atherosclerosis, and hyperlipidemia	Partial

Titchener et al. [24]	2013	Epicondylitis (lateral and medial)	Retrospective case-control, treatment study Level III	4998 versus 4998 controls (BMI calculated in 3,449 of cases (69%) and 3,049 (61.0%) of controls)	49 (interquartile range 42–56)	BMI	BMI > 40 and lateral epicondylitis OR: 1.41 (1.01–1.97) The effect disappeared when BMI was adjusted for consultation rate	Obesity is not associated with epicondylitis	No

Descatha et al. [28]	2013	Epicondylitis (lateral and medial)	Case-series (longitudinal study) Level IV	699 workers with no symptoms at baseline. At 36 months: 48 suffered from medial or lateral epicondylitis (6.9%), 34 from lateral epicondylitis (4.9%), 30 from medial epicondylitis (4.3%), and 16 from both	38.1 ± 9.3 (20–66)	BMI	BMI > 30 and lateral epicondylitis: univariate analyses OR: 2.4 (1.2–4.8), multivariate analyses OR: 1.8 (0.8–3.9). No association for medial epicondylitis	Obesity is associated only with lateral epicondylitis	Partial

Shiri et al. [20]	2006	Epicondylitis (lateral and medial)	Cross-sectional study (population study)	4,783 of the initial 5,871 (81.5%) 67 with lateral epicondylitis (1.3%) and 19 with medial epicondylitis (0.4%)	46.3 ± 9.6 (30–64)	BMI, waist circumference	Only in women WC > 100 cm and medial epicondylitis OR: 2.7 (1.2–6.0) BMI > 30 kg/m^2^ and medial epicondylitis OR: 1.9 (1.0–2.7) No association for lateral epicondylitis	Obesity is associated with medial epicondylitis.	Partial

Alvarez-Nemegyei [25]	2007	Pes anserinus	Retrospective case-control study Level III	22 cases of tendinopathy versus 38 controls	62.1 ± 11.5 for cases, 59.8 ± 9.4 for controls	BMI	Obesity: case 16/22 (72.7), controls 21/38 (55.3), *P* = .28 nonsignificant	No association	No

Taunton et al. [27]	2002	Patellar tendon	Retrospective case-control study Level III	96 cases versus 1906 controls	34.3	Weight, BMI	No association	No association	No

Frey and Zamora [21]	2007	Achilles, posterior tibial, and peroneal tendon	Cross-sectional study (population study)	1411 of which 208 with tendinitis/tendinosis	>18	BMI	123 (65.4%) of the overweight/obese subjects had a diagnosis of tendinitis compared to 65 (34.6%) normal subjects. BMI > 25 and tendinitis OR: 1.923 (1.39–2.66) *P* < .0001	Being overweight or obese significantly increased the chances of tendinitis	Yes

Holmes and Lin [26]	2006	Achilles	Retrospective case-control study, Level III	82 cases	49.5 (27–77)	BMI	Obesity was statistically associated with Achilles tendinopathy *P* = .025 for women and *P* = .001 for men, respectively	Obesity is one of the etiological factors of the Achilles tendinopathy	Yes

Gaida et al. [22]	2010	Achilles	Population-based study (cross-sectional study)	298 cases (127 men, 171 women) asymptomatic Achilles tendinopathy in 17 men (13%) and 8 women (5%) (*P* = .007).	Men 38.3 ± 12.2 Women 36.5 ± 10.5	Fat distribution (android/gynoid fat mass ratio and upper body/lower body fat mass ratio) determined using WC, WHR, and dual-energy X-ray absorptiometry	Men with Achilles tendinopathy had greater WHR (0.926 ± 0.091, 0.875 ± 0.065, *P* = .039), higher android/gynoid fat mass ratio (0.616 ± 0.186, 0.519 ± 0.142, *P* = .014), and higher upper body/lower body fat mass ratio (2.346 ± 0.630, 2.022 ± 0.467, *P* = .013). Women with tendinopathy had less total fat (17196 ± 3173 g, 21626 ± 7882 g, *P* = .009), trunk fat (7367 ± 1662 g, 10087 ± 4152 g, *P* = .003), and android fat (1117 ± 324 g, 1616 ± 811 g, *P* = .005). They had lower central/peripheral fat mass ratios (0.711 ± 0.321 g, 0.922 ± 0.194 g, *P* = .004) than women with normal tendons	Men with Achilles tendinopathy had a central fat distribution. Women had a peripheral fat distribution.	Yes

Scott et al. [15]	2013	Achilles	Frequency-matched case-control study Prognostic study, Level II	197 cases versus 100 controls	Cases: 52.77 ± 11.8 (21–82) Controls: 42.74 ± 12.1 (21–78)	BMI	Significant difference in BMI: *P* < .001 34.69 ± 7.54 (17.9–75.9) versus 30.56 ± 7.55 (19.7–61-5)	Patients with Achilles tendinopathy exhibited a significant higher BMI than controls	Yes

Klein et al. [16]	2013	Achilles	Frequency-matched case-control study Prognostic study, Level II	472 cases versus 472 controls	Cases: 51.2 ± 13.5 (16–88) Controls: 52.0 ± 14.3 (18–88)	BMI	OR: 2.60, 95% CI = 1.87–3.61; 3.81, 95% CI = 2.57–5.63; 3.77, 95% CI = 2.24–6.34; 6.56, 95% CI = 3.18–13.55 For BMI: 25.0–29.9, 30.0–34.9, 35.0–39.9, and >40.0, respectively	BMI plays a role in the development of Achilles tendinopathy	Yes

Taunton et al. [27]	2002	Achilles	Retrospective case-control study, Level III	96 cases versus 1906 controls	40.7	Weight, BMI	No association	No association	Np

Taunton et al. [27]	2002	Plantar fascia	Retrospective case-control study, Level III	158 cases versus 1846 controls	41.8	Weight, BMI	Weight >60 kg in female OR: 0.378 (0.203–0.706)	Women with a body weight greater than 60 kg were at increased risk of experiencing plantar fasciitis	Partial

Irving et al. [17]	2007	Chronic plantar heel	Frequency-matched case-control study Prognostic study, Level II	80 cases versus 80 controls	52.3 ± 11.7	BMI	Significantly greater BMI for CPHP group (29.8 ± 5.4 kg/m^2^ versus 27.5 ± 4.9 kg/m^2^; *P* < .01) CPHP were more likely to be obese (OR: 2.9, CI: 1.4–6.1, *P* < .01)	Obesity is associated with chronic plantar heel pain	Yes

Frey and Zamora [21]	2007	Plantar fascia	Cross-sectional study (population study)	1411 of which 189 with plantar fasciitis	>18	BMI	208 affected by plantar fasciitis BMI > 25 and plantar fasciitis OR: 1.4 (1.016–1.93) *P* < .040	If the subjects were overweight or obese, there was an increased likelihood, although not significant, of plantar fasciitis	No

Riddle et al. [18]	2003	Plantar fascia	Frequency-matched case-control study Prognostic study, Level II	50 cases versus 100 controls	49 ± 11 (31–85)	BMI	BMI > 30 OR: 5.6 (CI: 1.9–16.6) compared with the BMI ≤ 25 kg/m^2^	Obesity appears to be independent risk factor for plantar fasciitis.	Yes

BMI: body mass index; WC: waist circumference; WHR: waist-to-hip ratio; OG: group of obese patients; CG: control group; RC: rotator cuff; RCR: rotator cuff repair; CPHP: chronic plantar heel pain; OR: odds ratio; CI: confidence interval; and n.r.: not reported.
